# ALLOGENEIC PLATELET-RICH PLASMA FOR ROTATOR CUFF REPAIR

**DOI:** 10.1590/1413-785220172501163417

**Published:** 2017

**Authors:** CHRIS HYUNCHUL JO, JI SUN SHIN, SEUNG YEON LEE, SUE SHIN

**Affiliations:** 1. Seoul National University College of Medicine, SMG-SNU Boramae Medical Center, Department of Orthopedic Surgery, Seoul, Korea.; 2. Seoul National University College of Medicine, SMG-SNU Boramae Medical Center, Department of Laboratory Medicine, Seoul, Korea.

**Keywords:** Platelet-rich plasma, Rotator cuff, Growth factors, Tendon injuries

## Abstract

**Objective:**

To investigate the safety and efficacy of allogeneic platelet-rich plasma (PRP) in rotator cuff repair***.***

**Methods:**

Seventeen patients with a full-thickness rotator cuff tear were included. Ten patients underwent arthroscopic rotator cuff repair with allogeneic, and seven patients with autologous PRP. Three PRP gels in a volume 3 ml each were applied between the torn end and the greater tuberosity. Clinical outcomes were assessed preoperatively and at a minimum of 2 years after surgery. Structural outcomes were evaluated with the presence of retear and the change of the cross-sectional area (ACT) of the supraspinatus***.***

**Results:**

Allogeneic PRP did not cause any adverse events during the follow-up period. There was no significant difference in the clinical outcome measures between the two groups (all p > 0.05). The retear rate was 33.3% and 25.0% in the allogeneic group and autologous group, respectively (p = 0.764). The change between the one-year postoperative and immediately postoperative ACT was not also significantly different between the two groups (p = 0.373)***.***

**Conclusion:**

Allogeneic PRP in arthroscopic rotator cuff did not cause any local or general complications and that has the efficacy comparable to autologous PRP with respect to the clinical and structural outcomes.***Level of Evidence III, Retrospective Comparative Study.***

## RESUMO

**Objetivo:**

***Investigar a segurança e eficácia do plasma rico em plaquetas alogênico (PRP) no reparo do manguito rotador.***

**Métodos:**

***Foram incluídos dezessete pacientes com ruptura da espessura total do manguito rotador. Dez pacientes foram submetidos a reparo artroscópico do manguito rotador com PRP alogênico e sete pacientes com PRP autólogo. Três géis de PRP de 3ml cada foram aplicados entre a extremidade lesionada e a tuberosidade maior. Os resultados clínicos foram avaliados no pré-operatório e no mínimo 2 anos após a cirurgia. Os resultados estruturais foram avaliados com a presença de nova ruptura e a alteração da área em corte transversal (ACT) do supra-espinhal.***

**Resultados:**

***O PRP alogênico não causou quaisquer eventos adversos durante o período de acompanhamento. Não houve diferença significativa nas medidas de resultados clínicos entre os dois grupos (todos os valores p> 0,05). A taxa de nova ruptura foi de 33,3% e 25,0% no grupo alogênico e grupo autólogo, respectivamente (p = 0,764). A alteração da ACT entre o pós-operatório de um ano e imediatamente no pós-operatório também não foi significativamente diferente entre os dois grupos (p = 0,373) .***

**Conclusão:**

***O PRP alogênico administrado por via artroscópica no manguito rotador não causou quaisquer complicações locais ou gerais e sua eficácia é comparável ao PRP autólogo no que diz respeito aos resultados clínicos e estruturais. Nível de Evidência III, Estudo Retrospectivo Comparativo.***

## INTRODUCTION 

Platelet-rich plasma (PRP) has recently been actively investigated in various injuries and diseases.^1^ While the effects of PRP in arthroscopic rotator cuff repair have also been reported by several authors,^2^ ^-^ ^4^ the results are still not conclusive. Most of the previous studies used autologous PRP,^1^ ^,^ ^5^ ^,^ ^6^ but this plasma may not be available in certain patients such as patients with hematological diseases or who use anti-platelet medication, elderly patients with multiple comorbidities, patients who do not want to draw blood for any reason, and so on.^7^ ^,^ ^8^ Even though percutaneous local delivery of PRP improved fracture healing in diabetic rats,^9^ autologous PRP might not be an optimal solution since it has been reported that expression of platelet-derived growth factor decreased in diabetic animals.^10^ Therefore, an alternative to autologous PRP in these circumstances may be necessary, and allogeneic PRP is an option. Allogeneic platelets in the form of platelet concentrates have long been given safely in the treatment of thrombocytopenia or platelet dysfunction, or to patients with active platelet-related bleeding, or as prophylaxis in patients without cross-matching who are at serious risk of bleeding. Besides transfusion medicine, use of allogeneic PRP has only been described in a few case reports of oral and maxillofacial surgery.^8^ ^,^ ^11^ ^,^ ^12^ No study was found in the field of orthopedics except for one case report on the treatment of pseudoarthrosis of the distal tibia in a diabetic patient.^8^

The goal of this retrospective cohort study was to investigate the safety and efficacy of allogeneic PRP application in arthroscopic rotator cuff repair with regards to the clinical and structural outcomes. Our hypothesis was that allogeneic PRP was as safe and effective as autologous PRP. 

## PATIENTS AND METHODS

### Study design and patients

This retrospective cohort study was approved by the hospital's institutional review board (IRB of Seoul National University Boramae Medical Center, No. 262015112). We investigated clinical and structural outcomes in patients who underwent arthroscopic rotator cuff repair with allogeneic PRP in comparison with patients who received autologous PRP. All allogeneic PRP was obtained from patients who underwent arthroscopic rotator cuff repair with autologous PRP on the same day. The inclusion criteria were full-thickness rotator cuff tear treated by arthroscopic rotator cuff repair with allogeneic PRP and minimum follow-up of 24 months. 

From March 2009 to March 2011, 10 patients underwent arthroscopic rotator cuff repair with allogeneic PRP that was drawn and prepared from 8 patients who also underwent surgeries with autologous PRP on the same day; 4 with full-thickness rotator cuff tears and 3 with partial-thickness tears underwent rotator cuff repair, and 1 with a refracture of the clavicle and a metal failure received open reduction revision and internal plate fixation. Among the 10 patients who received allogeneic PRP, 6 received plasma from 6 different patients (5 with rotator cuff tears and 1 with a refracture), and the other 4 received plasma from 2 patients (2 from each one patient with a partial-thickness rotator cuff tear). Therefore, a total of 17 patients were included in the study: 10 in the allogeneic group, and 7 in the autologous group. 

### Preparation of PRP

PRP was obtained one day before surgery using a plateletpheresis system with a leukoreduction set (COBE Spectra LRS Turbo, Caridian BCT, Lakewood, Colorado) as previously described.^2^ An aliquot was used to determine complete blood counts using a fully automated analyzer (XE- 2100, Sysmex Corporation, Kobe, Japan), and concentration of fibrinogen was assessed using an automated coagulation analyzer (CA-7000, Sysmex Corporation). For use in the rotator cuff repair, the platelet counts in the PRP were adjusted with saline to 1000 x 10^3^ platelets per microliter. To produce a gel from the prepared PRP, 0.3 ml of 10% calcium gluconate was added to 3 ml of PRP. The dilution and gelling procedure was performed within one hour of surgical application.^2^

To assess the safety of the allogeneic PRP, tests for hepatitis B (HBV), hepatitis C (HCV), human immunodeficiency virus (HIV), and syphilis (VDRL) were performed and confirmed before application.^12^

### Surgical procedures and application of PRP

Arthroscopic surgery and application of PRP were performed with patients in the lateral decubitus position under general anesthesia, as described previously.^13^ ^,^ ^14^ Three PRP gels per patient were threaded to the suture and introduced into the 5.5 mm cannula after medial row sutures were placed. With the PRP gels in place, medial row sutures were tied using a SP knot if necessary.^15^ The lateral row was then secured using suture anchors, and the PRP gels were interposed at the tendon-bone interface. The patient's shoulders were immobilized for four to six weeks using an abduction brace and then passive range of motion (ROM) and active assisted ROM exercises were gradually permitted. Patients began strengthening exercises after three months.

### Outcome assessments

For the safety evaluation, general symptoms or signs related to immune responses such as fever, chills, pruritis, dyspnea, urticaria, or rash were observed. The wound site was also evaluated daily to determine the presence of zones of erythema, swelling, or abnormal discharge until patients were discharged from the hospital. 

Clinical outcomes were assessed according to: 1) pain, 2) ROM, 3) muscle strength, 4) overall satisfaction, and 5) functional scores. To evaluate structural integrity, magnetic resonance imaging (Achieva 3.0-T, Philips Medical System, Eindhoven, The Netherlands) with a dedicated shoulder coil was performed at least 9 months after surgery. Structural integrity was evaluated using Sugaya's MRI classification for patients.^16^ All images were reviewed by a fellowship-trained musculoskeletal radiologist and an orthopedic surgeon. The change in the cross-sectional area (CSA) of the supraspinatus was calculated by subtracting measures of the two different time points.^17^ ^-^ ^19^ The statistical analysis was performed using SPSS version 13.0 (SPSS Inc., Chicago, IL), and the significance level was set at p=0.05 throughout. 

## RESULTS 

### Baseline demographics & adverse events

All 17 patients were followed clinically for a minimum of 24 months. The average age was significantly higher in the allogeneic group (63.57 ± 9.61) than in the autologous group (56.33 ± 7.45), and there were more women in the allogeneic group (80.0%) than in the autologous group (28.6%). All patients in the allogeneic group had a full-thickness rotator cuff tear, while 3 of 7 (42.9%) in the autologous group had a partial-thickness rotator cuff tear (p = 0.023). The other characteristics did not differ significantly, as summarized in

Table 1.^20^ No general or local adverse events with regard to allogeneic or autologous PRP usage were observed during the immediate postoperative or follow-up periods.

**Table 1:** Baseline demographics.

	Allogeneic (n=10)	Autologous (n=7)	p-value
Mean age, y	63.57±9.61	56.33±7.45	0.029
Sex (male:female), n	2:8	5:2	0.034
Side (right:left), n	8:2	5:2	0.682
Dominance (yes:no), n	7:3	5:2	0.949
Duration, mo	14.40±13.02	21.71±19.46	0.366
Aggravation, mo	2.71±1.38	3.42±3.17	0.604
fRCT : pRCT, n	10:0	4:3	0.023
None: SLAP I : SLAP II+, n	7:2:1	6:0:1	0.450
SB tear grade (0:1:2:3)*, n	2:6:1:1	2:3:1:1	0.922
Biceps tear (none: partial : complete), n	6:3:1	3:3:1	0.784
Cofield type (P:S:M:L:MSV), n	0:1:5:3:1	3:0:1:1:2	0.103
Boileau stage (I:II:III:IV), n	4:1:4:1	3:1:1:2	0.614
Tendon grade (A:B:C)†, n	1:6:3	3:3:1	0.279
Treatment (StoS: 1 row : 2 row), n	1:1:8	2:1:4	0.556
Labrum (none : debride : repair), n	7:3:0	6:0:1	0.160
SB (none: debride : repair), n	3:5:2	2:2:3	0.551
Biceps (none: tenodesis : debride : tenotomy), n	7:0:3	4:1:2	0.464
Acromioplasty (yes:no), n	1:9	0:7	0.388
GT medialization (yes:no), n	1:9	2:5	0.323
GT coverage (A:B:C:D)‡, n	4:5:0:1	3:3:1:0	0.536
Fatty infiltration (0:1:2:3:4), n			
Supraspinatus	0:5:1:2:2	3:1:1:1:1	0.200
Infraspinatus	1:5:2:1:1	3:2:2:0:0	0.423
Subscapularis	4:6:0:0:0	3:3:0:1:0	0.435
GFID	1.43±0.88	1.05±0.80	0.370
Muscle atrophy			
Tangent (1:2:3)^§^, n	6:3:1	4:3:0	0.638
Occupation ratio (1:2:3)ǁ, n	4:4:2	4:1:2	0.519
Clinical follow-up, mo	29.57±5.97	28.29±5.59	0.946
MRI follow-up, mo	15.00±3.94	10.50±1.91	0.053

* Subscapularis tear was graded according to the Nove-Josserand et al.^20^ Grade 0, normal tendon; grade 1, tear less than one-quarter; grade 2, tear more than one-quarter but not complete; and grade 3, complete tear †Tendon grade assesses rotator cuff quality using three gross tendon criteria;^[14]^ (1) fraying over half of the tendon thickness; (2) delamination of the supraspinatus tendon, and (3) thinning of less than half of the normal thickness. A, none of these criteria were met; B, fraying or delamination was identified; and C, both fraying and delamination or thinning regardless of the other criteria ^‡^GT coverage evaluates the repair quality. A, complete coverage of the original footprint; B, incomplete coverage more than half of the footprint; C, incomplete coverage less than half of the footprint; and D, the presence of defect into the glenohumeral joint ^§^Tangent sign assesses muscle atrophy of the supraspinatus. Grade 1, negative, which means that the superior border of the supraspinatus was superior to the line tangential to the coracoid and scapular spine; grade 2, borderline, which means that the superior border was located about the tangential line; grade 3, positive, which means that the superior border was inferior to the tangential line. ^ǁ^Occupation ratio means the ratio of the CSA of the supraspinatus to the fossa. Grade 1, 0.6 to 1; grade 2, 0.4 to 0.6; grade 3, < 0.4. fRCT a full-thickness rotator cuff tear, pRCT a partial-thickness rotator cuff tear, SLAP II+ superior labral anterior and posterior lesion 2 and above, SB subscapularis, P:S:M:L:MSV small:medium:large:massive, StoS side-to-side repair, GT greater tuberosity, GFDI global fatty degeneration index

### Characteristics of PRP with respect to concentration, activation, and method of application

The characteristics of the PRP used in this study are described using the CAM classification.^21^ (Table 2) The average platelet count increased from 256.71 ± 72.04 (x 10^3^ platelets per microliter) in whole blood to 918.14 ± 149.61 in PRP, a 3.6-fold increase from the baseline (p < 0.001). The average red and white blood cell counts fell from 4.44 ± 0.48 to 0.15 ± 0.07 (x 10^3^ cells per microliter), and from 5.59 ± 0.68 in whole blood to 0.02 ± 0.01, respectively, representing a 0.03- and 0.004-fold decrease from the baseline, respectively (all p < 0.001). 

**Table 2:** Characteristics of the Platelet-Rich Plasma used in the study with respect to concentration, activation, and method of application

Concentration	Platelets, 10^3^/L	WBC, 10^3^/L	RBC, 10^3^/L	Fibrinogen, mg/dL
918.14 ± 149.61	0.02 ± 0.01	0.15 ± 0.07	NA
Activation	Activation status (%)*			Activation method	
	NA			calcium alone	
Method	State	Volume (ml)	Number	Interval (day)
gel	3x3	1	0

Data are expressed as average ± standard deviation in concentration properties. *Activation status was measured using flow cytometry with CD 61 and CD 62P in five patients. Results were expressed by the percentage of CD 62P-positive counts over CD 61-positive counts. WBC White blood cells, RBC Red blood cells, NA Not available

After surgery, all VAS pain measurements decreased significantly in both groups, and there were no significant differences between the two groups preoperatively or postoperatively. (Table 3) Representatively, average VAS pain measurements decreased from 5.41 ± 2.19 to 0.49 ± 0.69 in the allogeneic group (p <0.001), and from 3.50 ± 1.97 to 0.08 ± 0.17 in the autologous group (p= 0.010). 

**Table 3:** Pain, ROM, Strength and Overall Satisfaction

Variable	Allogeneic (n=10)			Autologous (n=7)			p-value
Pain at rest							
Preop	4.10 ± 1.66			3.33 ± 2.34			0.455
Final	0.29 ± 0.76			0.00 ± 0.00			0.219
p-value	<0.001			0.017			
Pain in motion							
Preop	6.33 ± 3.09			4.00 ± 2.37			0.136
Final	0.62 ± 0.73			0.10 ± 0.16			0.387
p-value	0.001			0.011			
Pain at night							
Preop	5.80 ± 2.78			3.17 ± 2.71			0.086
Final	0.57 ± 0.79			0.14 ± 0.38			0.331
p-value	0.001			0.048			
Pain average							
Preop	5.41 ± 2.19			3.50 ± 1.97			0.102
Final	0.49 ± 0.69			0.08 ± 0.17			0.317
p-value	<0.001			0.010			
Forward flexion, deg							
Preop	131.00 ± 36.73			165.71 ± 29.36			0.056
Final	175.00 ± 7.64			174.29 ± 7.87			0.960
p-value	0.005			0.448			
Abduction, deg							
Preop	122.00 ± 45.59			160.00 ± 44.35			0.108
Final	174.29 ± 15.12			177.14 ± 7.56			0.658
p-value	0.005			0.360			
External rotation arm at side, deg							
Preop	41.00 ± 15.06			45.71 ± 14.56			0.529
Final	46.43 ± 17.01			58.57 ± 16.26			0.102
p-value	0.635			0.022			
Internal rotation, vertebral level							
Preop	7.70 ± 3.86			9.14 ± 3.53			0.445
Final	11.57 ± 1.40			10.86 ± 1.57			0.628
p-value	0.020			0.127			
Supraspinatus, lb							
Preop	4.12 ± 3.03			6.46 ± 3.34			0.154
Final	8.06 ± 2.60			9.31 ± 4.47			0.679
p-value	0.016			0.128			
Infraspinatus, lb							
Preop	6.80 ± 3.02			6.41 ± 2.96			0.797
Final	7.93 ± 2.05			9.37 ± 4.55			0.421
p-value	0.318			0.088			
Subscapularis, lb							
Preop	6.10 ± 2.37			8.77 ± 5.11			0.165
Final	11.29 ± 3.77			13.90 ± 5.84			0.200
p-value	0.001			0.138			
Surgery again, n (%)							
Final	8 (80.0)			6 (85.7)			0.761
Recommend surgery, n (%)							
Final	8 (80.0)			7 (100.0)			0.208
Overall function							
Preop	4.30 ± 2.36			5.67 ± 1.21			0.212
Final	8.57 ± 1.51			9.10 ± 1.18			0.199
p-value	<0.001			0.001			
Overall satisfaction							
Final	88.57 ± 13.45			92.14 ± 9.06			0.353

Forward flexion, abduction, and internal rotation significantly increased while external rotation of the arm at the side did not increase in the allogeneic group. (Table 3) In the autologous group, external arm rotation at the side significantly increased, while forward flexion, abduction, and internal rotation did not. No significant differences were found between the two groups for active forward flexion, abduction, external arm rotation at the side, and internal rotation before and after surgery. 

Supraspinatus strength improved significantly in the allogenic group, from 4.12 ± 3.03 lb to 8.06 ± 2.60 lb (p= 0.016), but this was not the case for the autologous group, which showed a less notable improvement from 6.46 ± 3.34 lb to 9.31 ± 4.55 lb (p= 0.128). (Table 3) Infraspinatus strength did not significantly improve in either groups, increasing only slightly from 6.80 ± 3.02 lb to 7.93 ± 2.05 lb in the allogeneic group (p = 0.318) and from 6.41 ± 2.96 lb to 9.37 ± 4.55 lb in the autologous group (p= 0.088). Subscapularis strength improved significantly from 6.10 ± 2.37 lb to 11.29 ± 3.77 lb in the allogeneic group (p= 0.001), but not in the autologous group, which increased from 8.77 ± 5.11 lb to 13.90 ± 5.84 lb (p = 0.138). The strength of the supraspinatus, infraspinatus, and subscapularis did not differ significantly in the two groups preoperatively or postoperatively. 

Eight out of 10 patients (80.0%) in the allogeneic group expressed willingness to undergo surgery again and wouldrecommend the surgery to other patients; in the autologous group this number was 6 (85.7%) and 7 (100.0%) out of 7 patients, respectively, showing no difference between the two groups. (Table 3) Overall function improved from 4.30 ± 2.36 to 8.57 ± 1.51 in the allogeneic group (p < 0.001), and from 5.67 ± 1.21 to 9.10 ± 1.18 in the allogeneic group (p = 0.001). Overall satisfaction was 88.57 ± 13.45 in the allogeneic group, and 92.14 ± 9.06 in the autologous group.

ASES, Constant, DASH, SST, and SPADI scores significantly improved after surgery, while the UCLA score did not improve in either group. (Table 4) No significant differences were found in all the scores between the two groups both preoperatively and postoperatively. 

**Table 4:** ASES, Constant, UCLA, DASH, SST, and SPADI Scores.

Variable	Allogeneic (n=10)			Autologous (n=7)			p-value
ASES							
Preop	42.28 ± 23.22			63.06 ± 17.49			0.080
Final	92.77 ± 6.86			98.65 ± 2.00			0.228
p-value	<0.001			0.005			
Constant							
Preop	43.32 ± 19.21			56.70 ± 12.35			0.152
Final	76.35 ± 7.52			82.03 ± 4.96			0.162
p-value	0.001			0.001			
UCLA							
Preop	26.43 ± 18.27			19.97 ± 6.15			0.421
Final	32.29 ± 3.50			28.57 ± 12.93			0.808
p-value	0.868			0.256			
DASH							
Preop	49.75 ± 27.16			31.19 ± 23.97			0.167
Final	6.19 ± 6.65			1.67 ± 2.31			0.190
p-value	0.002			0.017			
SST							
Preop	5.10 ± 3.38			7.33 ± 3.44			0.225
Final	11.29 ± 1.25			11.86 ± 0.38			0.263
p-value	0.001			0.017			
SPADI							
Preop	54.91 ± 28.62			37.10 ± 23.13			0.219
Final	6.14 ± 5.65			0.99 ± 1.81			0.308
p-value	0.001			0.015			

Data are expressed as mean ± standard deviation.

Postoperative MRI or CTA was performed in 14 of 17 patients in the study (82.4%): 10 patients (100.0%) in the allogeneic group, and 4 patients (57.1%) in the autologous group. (Table 5) The mean MRI follow-up was 15.00 ± 3.94 months (range 9-20) in the allogeneic group and 10.50 ± 1.91 months in the autologous group. One patient in the allogeneic group was excluded from analysis of structural integrity because rotator cuff repair was done only partially because of limited excursion. Therefore, 9 patients in the allogeneic group and 4 patients in the autologous group were included in the analysis. The retear rate did not differ significantly between the two groups: 33.3% (3 of 9) in the allogeneic group, and 25.0% (1 of 4) in the autologous group (p = 0.764).

**Table 5:** Retear Rates.

	Allogeneic (n=9)	Autologous (n=4)	p-value
Retear	3 (33.3%)	1 (25.0%)	0.764

Data are expressed as n (%). PRP, Platelet-Rich Plasma

Supraspinatus CSA taken immediately after surgery and one-year postoperative differed between the two groups; (Table 6) The CSA in the autologous group were greater than those in the allogeneic group at each time point. However, the change between the one-year postoperative and immediate postoperative CSA was not significantly different between the two groups: -67.42 ± 58.75 mm^2^ in the allogeneic group, and -49.05 ± 58.57 mm^2^ in the conventional group (p = 0.373).

**Table 6:** Change in the Cross-Sectional Area (CSA) of the Supraspinatus 1 Year after Rotator Cuff Repair.

Cross-sectional area, mm²	Allogeneic (n=9)	Autologous (n=4)	p-value
Immediately postoperative	376.77 ± 63.53	478.66 ± 99.63	0.045
One-year postoperative	309.35 ± 69.04	429.61 ± 108.45	0.032
Δ(PO1yr-ImPO)*	-67.42 ± 58.75	-49.05 ± 58.57	0.373

Data are expressed as mean ± standard deviation. *Δ(PO1yr-ImPO), a change of the cross-sectional area of the supraspinatus between the 1-year postoperative measure and immediate postoperative measures.

## DISCUSSION 

This study showed that allogeneic PRP application in arthroscopic rotator cuff repair did not cause any adverse events, but demonstrated efficacy in clinical and structural outcomes comparable with autologous PRP for the first time, to our knowledge. While many studies have been performed to determine the effect of PRP in various injuries and diseases,^1^ ^,^ ^2^ ^,^ ^22^ most used autologous plasma, except for a few case reports.^7^ ^,^ ^8^ ^,^ ^11^ ^,^ ^23^Except for one *in vitro* study which investigated the effect of allogeneic PRP compared to autologous PRP,^24^ no clinical studies have compared the safety and efficacy of allogeneic PRP over autologous PRP. Therefore, based on findings of this study, we suggest that allogeneic PRP may be a viable alternative when autologous PRP is not available or is less appropriate. However, before allogeneic PRP is used widely in the field of regeneration medicine, we also suggest that safety issues including bacterial or viral disease transmission and immune reaction be further clarified to guarantee patient safety, even though platelet transfusion via platelet pheresis is a safe and standard method undertaken within a highly-developed regulatory environment in blood donation services. For example, the use of a leukoreduction set may be an option, as in the current study,^8^ and some kinds of pathogen reduction technologies may merit consideration.^25^

An important cause of controversial results of PRP in rotator cuff repair derives from the wide variability of PRP used in these studies,^26^ resulting from different concentrations of cells, activation status, and application method through different preparation systems. Therefore, if these differences could be removed or avoided the real effects of PRP in rotator cuff repair could be more clearly elucidated. In this sense, allogeneic PRP may have several advantages over autologous PRP. First, as a standard blood bank product, a single-donor PRP is very easy to obtain, safe, and would be available in large quantities and incur less cost.^8^ ^,^ ^11^ ^,^ ^27^ Second, the collection process for this product is well-established and highly standardized with regard to the use of anticoagulant, separation and processing techniques, centrifugal force, and temperature and time, resulting in highly predictable amount of platelets, white blood cells, red blood cells, and fibrinogen. Third, standardized preparation process and products may allow researchers to investigate the effects of PRP more clearly, and the results from various studies could be compared more easily. Additionally, a more standardized method of obtaining allogeneic PRP, such as the platelet pheresis used in this study, would cause less activation of platelets during preparation than manual separation methods.^27^ ^,^ ^28^ Finally, the PRP could be prepared from younger patients in better health. Taken together, allogeneic PRP may have a great potential as a useful tool for biologic treatment strategies and could prove valuable clues to understand the mechanism of PRP, which has not yet occurred with autologous plasma. 

One of the strengths of this study was that the patients in the comparison group were the corresponding donors of allogeneic PRP who also underwent the same surgery on the same day. This setting of the study eliminates some concerns about the potential variability of allogeneic PRP, surgical techniques, etc., thus making the results of the study more valuable. Another strength was that the allogeneic PRP was not manufactured and stored for a certain time, but freshly manufactured one day prior to surgery and was used the next day during the procedure, just like autologous PRP. This might be also helpful for removing any possible effects of storing PRP. 

Limitations of the present study include; 1) small number of relatively heterogenic participants with regard to age, sex, and patients with partial-thickness rotator cuff tear in the control group, which may increase the risk of a type II error, 2) a lack of randomization, 3) incomplete characterization of PRP, such as absence of activation level or concentration of important growth factors, 4) low MRI follow-up rate to assess retearing, and 5) potential risk of adverse events related to transmission of viral and non-viral immunological infections despite HBV, HCV, HIV, and VDRL screening and use of a leukoreduction set. 

## CONCLUSION

Allogeneic PRP in arthroscopic rotator cuff repair did not cause any local or general complications and has an efficacy comparable to autologous PRP with respect to clinical and structural outcomes. This study provides the first evidence of the safety and efficacy of allogeneic PRP in arthroscopic rotator cuff repair. Further randomized clinical trials should be performed to support this preliminary study of allogeneic PRP for rotator cuff repair.
